# Ink-Deposited Transparent Electrochromic Structural Colored Foils

**DOI:** 10.1021/acsami.2c11106

**Published:** 2022-08-19

**Authors:** Arne A. F. Froyen, Nadia Grossiord, Jos de Heer, Toob Meerman, Lanti Yang, Johan Lub, Albert P. H. J. Schenning

**Affiliations:** † Stimuli-Responsive Functional Materials and Devices, Department of Chemical Engineering and Chemistry, Eindhoven University of Technology, P.O. Box 513, 5600 MB Eindhoven, The Netherlands; ‡ Institute for Complex Molecular Systems, Eindhoven University of Technology, Den Dolech 2, 5600 MB Eindhoven, The Netherlands; § SABIC, Plasticslaan 1, 4612 PX, Bergen op Zoom, The Netherlands; ∥ SCNU-TUE Joint Laboratory of Device Integrated Responsive Materials (DIRM), South China Normal University, Guangzhou Higher Education Mega Center, 510006 Guangzhou, China

**Keywords:** structural color, transparent heater, silver nanowire, cholesteric liquid crystal, photonic coating, electrochromic

## Abstract

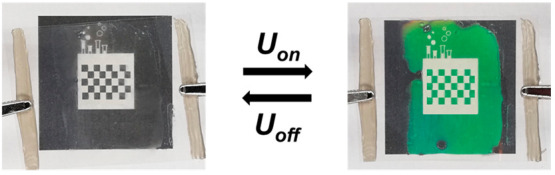

Despite progress in the field of electrochromic devices, developing structural color-tunable photonic systems having both high transparency and flexibility remains challenging. Here, an ink-deposited transparent electrochromic structural colored foil displaying reflective colors, tuned by an integrated heater, is prepared in a single-substrate method. Efficient and homogeneous heating is induced by a gravure printed silver nanowire-based substrate, delivering an electrothermal response upon applying an electrical potential. On top of this flexible, transparent heater, a cholesteric liquid crystal ink is bar-coated and subsequently photopolymerized, yielding a structural colored film that exhibits temperature-responsive color changes. The transparent electrochromic foils appear colorless at room temperature but demonstrate structural color tuning with high optical quality when modifying the electrical potential. Both optical and electrothermal performances were preserved when deforming the foils. Applying the conductive and structural colored inks via the easy processable, continuous methods of gravure printing and bar-coating highlights the potential for scaling up to large-scale stimuli-responsive, transparent optical foils. These transparent structural colored foils can be potentially used for a wide range of photonic devices including smart windows, displays, and sensors and can be directly installed on top of curved, flexible surfaces.

## Introduction

Temperature-responsive optical devices based on pigments or structural color can alter their color upon exposure to a thermal stimulus and have illustrated their potential for implementation in various applications including sensors, smart windows, and anticounterfeit labels.^1−5^ Although the optical properties are autonomously altered upon thermal fluctuations, manipulating the response by electricity could lead to more user-friendly devices. Integrating a heating element to a thermosensitive system introduces an electrothermal stimulus, generating a temperature increase when an electric current or potential is applied to the conductive system.^6^ The amount of heat generated depends on the electric current flowing across the conductor, allowing for precise temperature tuning.^7^ In contrast to uniform color tuning when using a continuous film heater, patterned colors appear when locally heating a thermochromic system using patterned electrodes.^8−13^ Despite the promising studies on electrothermally driven photonic devices, a major challenge in this field is the development of simultaneously flexible and transparent photonic systems with multicolor tuning, vitally important for effective display and window applications. Most reports on electrochromic devices make use of temperature-responsive pigments, which can only be switched between colored and discolored states when applying an electrothermal stimulus and are limited to nontransparent devices due to their low optical quality.^8,14−18^ Recent progress has led to the realization of electrothermally driven structural color-tunable systems, but these are limited to either rigid transparent^7,19−22^ or flexible nontransparent^23−27^ devices due to the poor mechanical or optical properties of at least one layer in the final material. Additionally, scaling up to large-area applications remains rather difficult since scalable processes to develop such multilayered photonic foils have been rarely reported.^4,26,28^ To establish an electrothermally driven structural colored system featuring both high transparency and flexibility, a flexible, transparent heater must be integrated.

Transparent heaters consist of thin, electrically conductive layers that efficiently induce rapid, controllable Joule heating while applying an electric current or potential over the surface area.^29^ Currently, transparent conductive oxides, especially indium tin oxide, are the most prominent conductive materials for manufacturing transparent heaters but are restricted in use due to their limited mechanical flexibility. Intensive research has led to the development of a wide variety of electrically conductive materials that may be utilized as flexible transparent heaters.^30^ Silver nanowires (AgNWs) have attracted particular interest since they combine high optical transparency and flexibility with a low sheet resistance when forming a percolating network.^31−33^ Furthermore, flexible transparent heaters can be prepared from conductive ink formulations of metallic nanowires dispersed in a solvent that can be applied on top of flexible substrates via spray coating,^34,35^ screen printing,^36,37^ and inkjet printing,^38^ among other techniques. To establish uniform, energy-efficient heating, good adhesion between the substrate and nanowires is required. Due to the low adhesion of AgNWs, lamination could result in nonuniform heating in the final device. Adding a polymer material to the conductive ink forms an encapsulating film after deposition that facilitates uniform heating.^30^ In addition, embedding the heater is more beneficial to establish proper adhesion and improves the mechanical properties of the photonic device compared to a laminated system.^39^

Cholesteric liquid crystals (CLCs) are frequently used to achieve structural color as they selectively reflect a specific handedness of circularly polarized light. The central wavelength of the reflection band is dependent on the pitch length, which is defined by the periodicity of the underlying helical structure of the liquid crystal molecules. When using temperature-responsive CLC mixtures, the initial reflective color can be altered through a reduction or increase of the pitch length, caused by a reorganization of the helices upon heating and cooling.^5,40,41^ In general, robust CLC coatings are obtained by incorporating the liquid crystal mesogens into a cross-linked polymer network.^42−44^ Despite the improved mechanical robustness, a highly cross-linked CLC system is almost temperature-independent, making them unsuitable for thermochromic color-tunable devices. To realize a polymerized photonic coating while retaining the characteristic thermal response of the small mesogenic CLC molecules, a nonreactive, thermosensitive CLC fraction should be encapsulated by a polymer matrix. Such structural colored coatings were obtained out of a homogeneous mixture via directionally controlled photoinduced phase separation (PIPS), known as photo-enforced stratification, yielding a flexible single-substrate device that displayed rapid and large color tuning upon body temperature exposure.^45−47^ During stratification, the reactive monomers, which were present in the initial homogeneous mixture, form an acrylic polymer that becomes immiscible with the nonreactive liquid crystalline fraction over time, resulting in phase separation of these components.^48,49^ Eventually, a hard polymer topcoat is formed, which protects the fluidlike, nonreactive CLC fraction underneath. When using a conductive foil as the bottom substrate, electrothermally driven color tuning should be observed for these stratified reflective coatings.

In this work, we present highly flexible, transparent heaters developed by depositing an AgNW-based ink over a PET substrate via gravure printing with rapid and homogeneous heating over the entire surface area when a low electrical potential is applied, which is preserved upon bending. On top of this conductive substrate, a stratified temperature-responsive photonic coating was formed out of a CLC ink after bar coating, displaying multicolor tuning upon heating. The final transparent photonic foil showed rapid and reversible electrothermal color tuning over the entire visible region when modifying the electrical potential. Even when repetitively deforming the foil to large bending angles, the reflective color could be preserved without damaging either the coating or the transparent heater, showing its flexibility. In addition, structural colored foils are realized, using continuous solution-processed roll-to-roll methods, highlighting their potential in a variety of stimuli-responsive photonic devices ranging from small-scale sensors or anticounterfeit labels to large-area applications such as displays or smart windows.

## Results and Discussion

**Figure 1:**
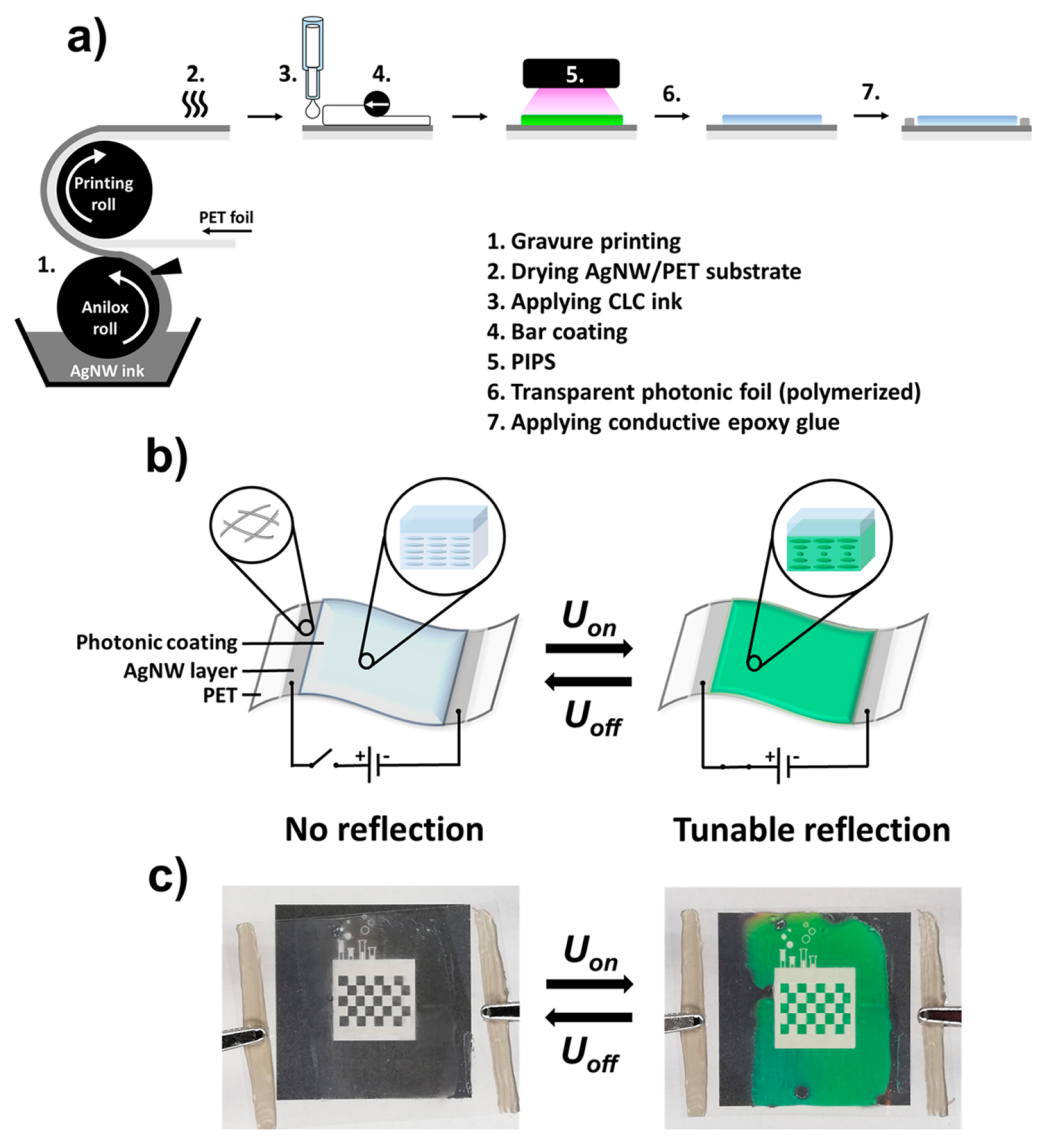
(a) Overview of the procedure to obtain the electrothermally driven structural colored foils. (b) Schematic representation of the electrochromic structural colored foil. A flexible, transparent AgNW/PET heater induces reflection band shifting under electrical stimulation. In the absence of an electrical stimulus (*U*_off_), the transparent foil remains colorless at room temperature, while a reflective color can be displayed upon applying an electrical potential (*U*_on_). (c) Images of the actual device (4 × 4.5 cm^2^) showing high optical quality and a reflective color when applying *U* = 3.5 V. The initial state is regained after removing the electrical stimulus.

Continuous roll-to-roll processes (Figure 1a) were used to develop a transparent electrochromic foil by first applying an AgNW-based ink on top of a transparent PET substrate via gravure printing (step 1). Subsequent drying created the thin film heater (step 2). On top of the printed AgNW/PET substrate, a temperature-responsive photonic coating was formed via PIPS after bar coating a CLC ink over the AgNW/PET substrate (steps 3–5), yielding a transparent photonic foil that appeared colorless at room temperature (step 6). Afterward, a conductive epoxy glue was applied on top of the AgNW/PET heater to induce proper electrical contact (step 7).

Reversible electrochromic color tuning was presented when applying a low potential (*U*) to the conductive substrate (Figure 1b). Joule heating was induced due to the percolating network of the AgNWs deposited on the PET substrate. Accordingly, an adjustable electrothermal stimulus could be applied to the photonic foil. This caused the appearance of a tunable reflective color in the temperature-responsive photonic coating, consisting of a thin thermosensitive CLC layer that was protected by a hard polymerized topcoat. Images of the actual device showed that structural color could be visualized with high optical quality, i.e., minimal observed absorption or scattering, making these foils suitable for transparent applications (Figure 1c).

**Figure 2:**
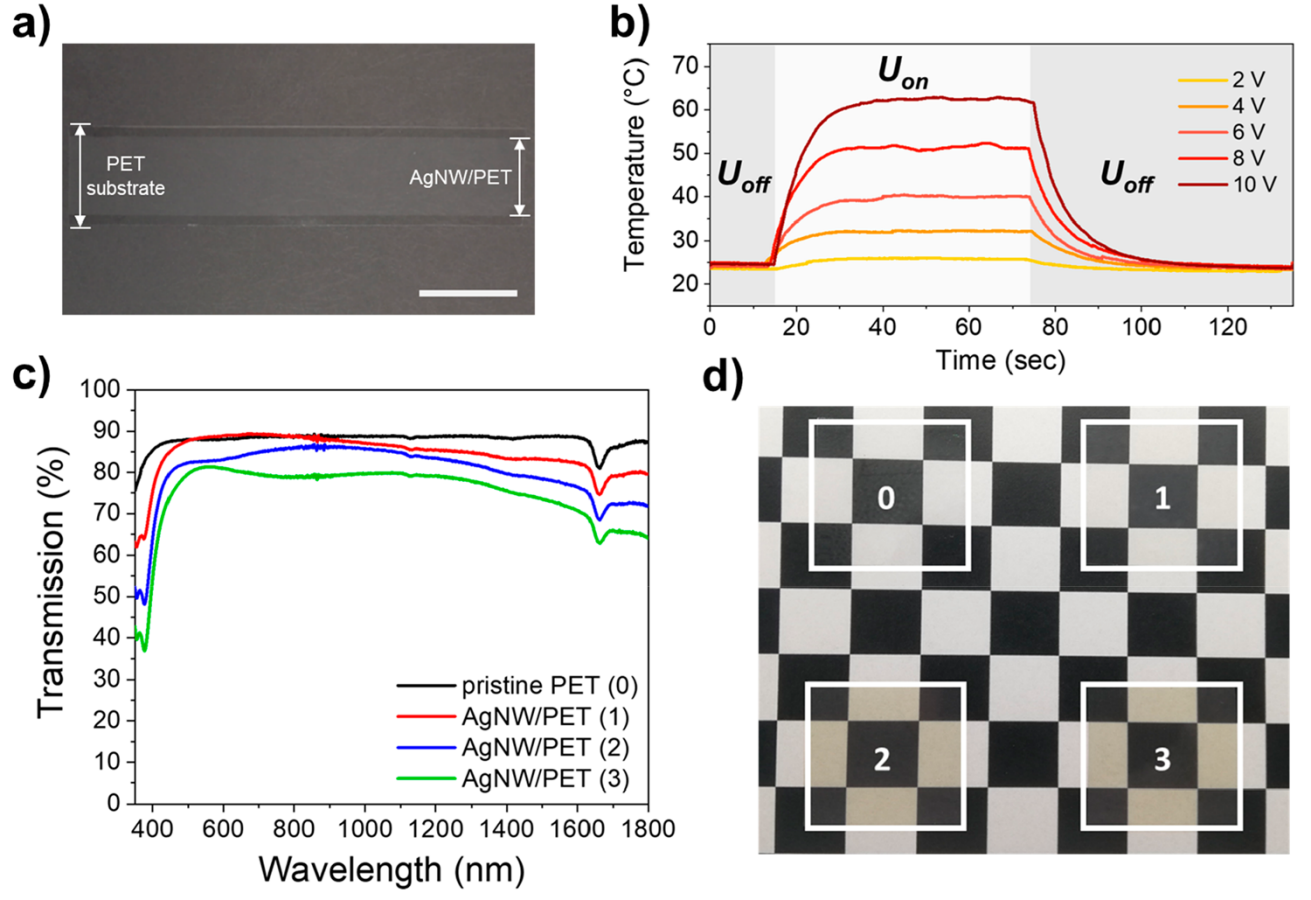
(a) Image of a large-area, transparent AgNW/PET heater (foil dimensions: 24 × 5 cm^2^) obtained via gravure printing (scale bar = 5 cm). The width of the PET foil and AgNW/PET heater are indicated in the image, showing that the edges of the PET foil were not covered by the conductive ink during printing due to the limited width of the anilox roll. (b) Time-dependent temperature profiles of the electrothermal response of the AgNW/PET substrate when changing the electrical potential. Joule heating was generated when applying a potential (*U*_on_) at *t* = 15 s, reaching a steady-state temperature (*T*_ss_) until the electrical stimulus was removed (*U*_off_) at *t* = 70 s. (c) Transmission spectra of a pristine PET foil (0) and AgNW/PET heaters printed one (1), two (2), or three (3) times with the conductive ink. (d) Images of the PET foils (3 × 3 cm^2^) measured in (c) on top of a black and white background. The numbers correspond to the number of printed layers as is indicated in Figure 2c.

A conductive ink, consisting of AgNWs dispersed in a solvent, was chosen since AgNW-based heaters can be produced with a high figure of merit due to the presence of a percolation network.^50^ Gravure printing was exploited to transfer the conductive ink to the substrate, resulting in a controlled deposition of AgNWs over the surface of the PET foil (Figure 2a). The solvent was evaporated, resulting in the formation of a thin AgNW film that functioned as the conductive layer. Unless stated otherwise, smaller pieces (4 × 4.5 cm^2^) were cut from the printed AgNW/PET strips that were used in further experiments. The sheet resistance (*R*_s_) of the AgNW/PET foils was determined using a four-point probe method: printing a single layer of the AgNW-based ink on top of the substrate resulted in a transparent heater with *R*_s1_ = 25.84 ± 0.35 Ω sq^–1^; high conductivity was achieved after a single printing step. Conductive epoxy glue was applied over the width of the conductive substrate on both edges to ensure good electrical contact. To verify whether efficient Joule heating could be induced by these AgNW/PET foils, the heating capability was studied upon applying an electrical potential. As soon as the electrical input was provided (*U*_on_), Joule heating was induced by the AgNW/PET foil (Figure 2b), causing a temperature increase until a steady-state temperature (*T*_ss_) was reached, which was maintained until the applied potential was switched off (*U*_off_) again. The time–temperature profiles showed a rapid electrothermal response, reaching *T*_ss_ in *t* < 20 s, while cooling down to room temperature was achieved in *t* < 30 s. The amount of heat generated by the AgNW/PET substrate could be modulated by varying *U*, meaning that *T*_ss_ could be easily adjusted. Uniform temperature distribution was observed for the transparent heater thereby confirming a homogeneous AgNW distribution over the printed surface area (Figure S1). In addition, the flexibility of these printed AgNW/PET foils was tested by monitoring the resistance under repetitive bending (Figure S2). No deviation of the initial resistance was detected over multiple bending cycles, confirming our claim that a flexible transparent heater was obtained.

The electrical properties of these transparent heaters could readily be tweaked by changing the printing procedure. As an example, a PET foil was repeatedly printed with the AgNW-based ink under identical printing conditions to investigate the effect of multiple printing layers on the electrical conductivity. After each printing step, the foil was left to dry before applying a subsequent layer. Eventually, sheet resistances *R*_s2_ = 11.78 ± 0.18 Ω sq^–1^ and *R*_s3_ = 7.41 ± 0.04 Ω sq^–1^ were obtained for a PET foil undergoing two or three printing steps, respectively. Despite the improved conductivity, increasing the number of printing layers has a negative impact on the optical transparency of the conductive foils (Figure 2c,d), stemming from the increased AgNW density on top of the surface of the PET foils (Figure S3).^50,51^ Hence, transparent AgNW/PET substrates that underwent a single printing step were used for further experiments.

**Figure 3:**
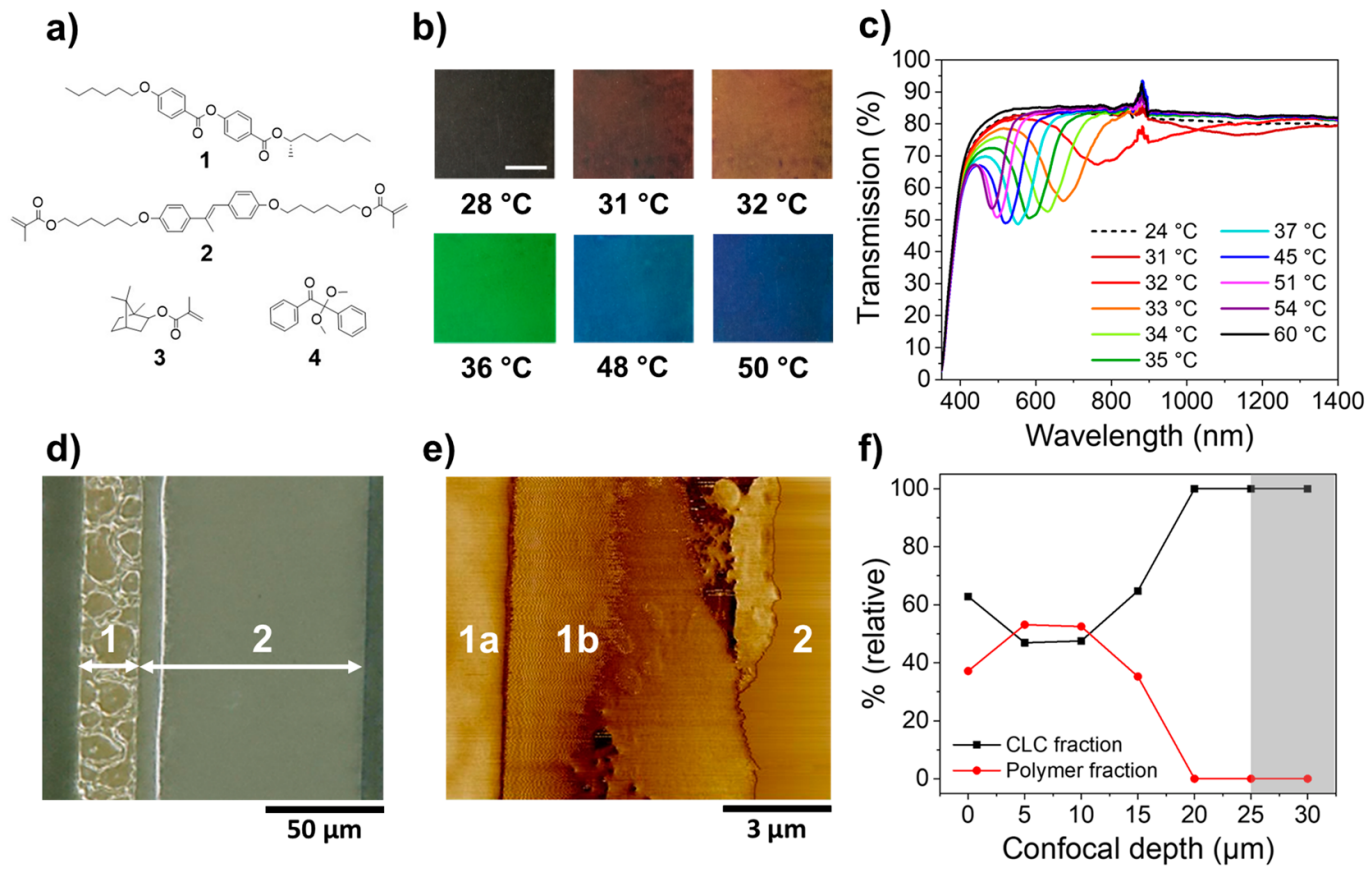
(a) Chemical composition of the mixture used in this study. The mesogenic mixture MLC-2138 was omitted as the molecular structures are unknown. (b) Images of the stratified structural colored coating on top of an AgNW/PET substrate upon heating (scale bar = 1 cm). A black background was used to improve color contrast. (c) Transmission spectra showing the temperature-responsive reflection band shift of the photonic coating on top of the AgNW/PET substrate. (d) Optical microscopy image of a cross section of the photonic coating (1) on top of the AgNW/PET (2) substrate. (e) AFM image of the same cross section near the coating-AgNW/PET interface. Below the polymerized topcoat (1a), a thin CLC layer (1b) was observed next to the substrate (2). (f) Relative ratio between the CLC and polymer fraction measured through the coating thickness by confocal Raman spectroscopy. The coating (white area) was roughly 25–30 μm as can be confirmed from the optical microscopy image. The gray area corresponds to the AgNW/PET layer.

To realize electrothermal color tuning, a temperature-responsive photonic coating was formed directly on top of the transparent AgNW/PET heater. PIPS was exploited to obtain a stratified structural colored coating.^45,46,48^ A temperature-responsive CLC mixture exhibiting a smectic–cholesteric phase transition (Figures 3a and S4) consisting of a nematic liquid crystalline mixture (MLC-2138) and a chiral dopant (**1**) was used, as has been reported earlier.^46,52,53^ A novel stilbene-derived diacrylate (**2**) was synthesized, and together with a monoacrylate (**3**) and photoinitiator (**4**) added to the thermosensitive mixture to form the rigid polymer topcoat. The CLC ink was bar coated over the AgNW/PET substrate, and subsequent UV illumination induced PIPS in the wet film. The stilbene-derived diacrylates stimulated proper layer formation during stratification. During photopolymerization, diffusion of reactive stilbene diacrylates toward the light source improves phase separation, while additionally a UV intensity gradient is created since the stilbene group absorbs in the UV region.^48,49^ The result was a transparent photonic coating that appeared colorless at room temperature.

To investigate the thermochromic behavior of the foil, the sample was first heated on a hot plate, which resulted in the appearance of a homogeneous reflective color that could be shifted over the entire visible region at elevated temperatures (Figure 3b,c). Like the pure CLC mixture, the optical response of the photonic coating stemmed from a smectic–cholesteric phase transition (Figure S5). At room temperature, the liquid crystals were ordered in a smectic phase; hence, no reflection band was recorded. However, heating induced a transition to the cholesteric phase, resulting in the reflection of visible light. Further heating of the coating enacted a blueshift of the reflection band over the entire visible region until the isotropic state was reached around *T* = 60 °C. The transmission spectra showed high transmission values in the visible region, suggesting planar alignment of the helices inside the phase-separated photonic coating since no indications of refractive index mismatches were detected, which could cause scattering. Additionally, it can be concluded that by reaching the reflection limit of 50% for CLCs, a significant number of stacked helices (pitches) should be present after PIPS, indicating a significant degree of phase separation between the polymer network and the nonreactive CLC fraction. The printed AgNW layer did not influence the thermal response of the photonic layer as an identical reflection band shift was detected for a phase-separated photonic coating on top of a pristine PET substrate (Figure S6). Furthermore, the optical response is similar as no significant differences in the positions of the reflection band were detected between the samples under isothermal conditions, implying that the phase separation behavior was not influenced by the presence of the conductive AgNW layer.

After photopolymerization, cross sections of the foil were analyzed with optical microscopy, revealing distinct domains throughout the coating thickness, indicating that phase separation had occurred between the polymer and nonreactive CLC fraction (Figure 3d). The thickness of the PET substrate was confirmed to be approximately 100 μm, while the coating thickness was roughly 25–30 μm. The observed white line in the PET substrate could be attributed to liquid crystal penetration, causing a local refractive index mismatch.^54^ Capturing sharp images at higher magnification turned out to be unsuccessful since the foil moved during the measurements, suggesting fluidlike behavior inside the coating. Atomic force microscopy (AFM) was employed to study the foil near the PET interface with a higher resolution (Figure 3e). Adjacent to a polymerized layer (1a), a thin layer (1b) was observed that showed vibration features with frequencies dependent on the scanning speed. Such features can be ascribed to layers that can locally move or flow during the measurement, implying the presence of a nonpolymerized CLC layer of roughly 3–8 μm. The bordering layer (1a) observed on top of this fluidlike layer should consist of both polymer and CLC material. These observations were supported by confocal Raman spectroscopy, analyzing the relative ratio of the CLC to polymer fraction throughout the coating thickness (Figure 3f). To determine the coating composition, the ratio between the peak area 2190–2250 cm^–1^ (corresponding to the −CN functional groups in the CLC mixture) and the peak area 2848–3028 cm^–1^ (corresponding to the CH_2_ of monoacrylate **3**) was examined throughout the coating thickness (Figure S7).^45^ An equal contribution of the CLC and polymer fraction was observed inside the coating, but near the PET surface, the CLC fraction became the dominant fraction. The printed AgNW layer could not be directly observed with these techniques, indicating this layer is only a few nanometers thick and cannot be visualized due to the limited resolution of the analytical techniques. Based on the combined microscopy and Raman analyses, it appears that a phase-separated photonic coating was obtained, composed of an unpolymerized CLC layer (3–8 μm) that was covered by a rigid topcoat (20–25 μm) containing CLCs encapsulated by a cross-linked polymer network.

**Figure 4:**
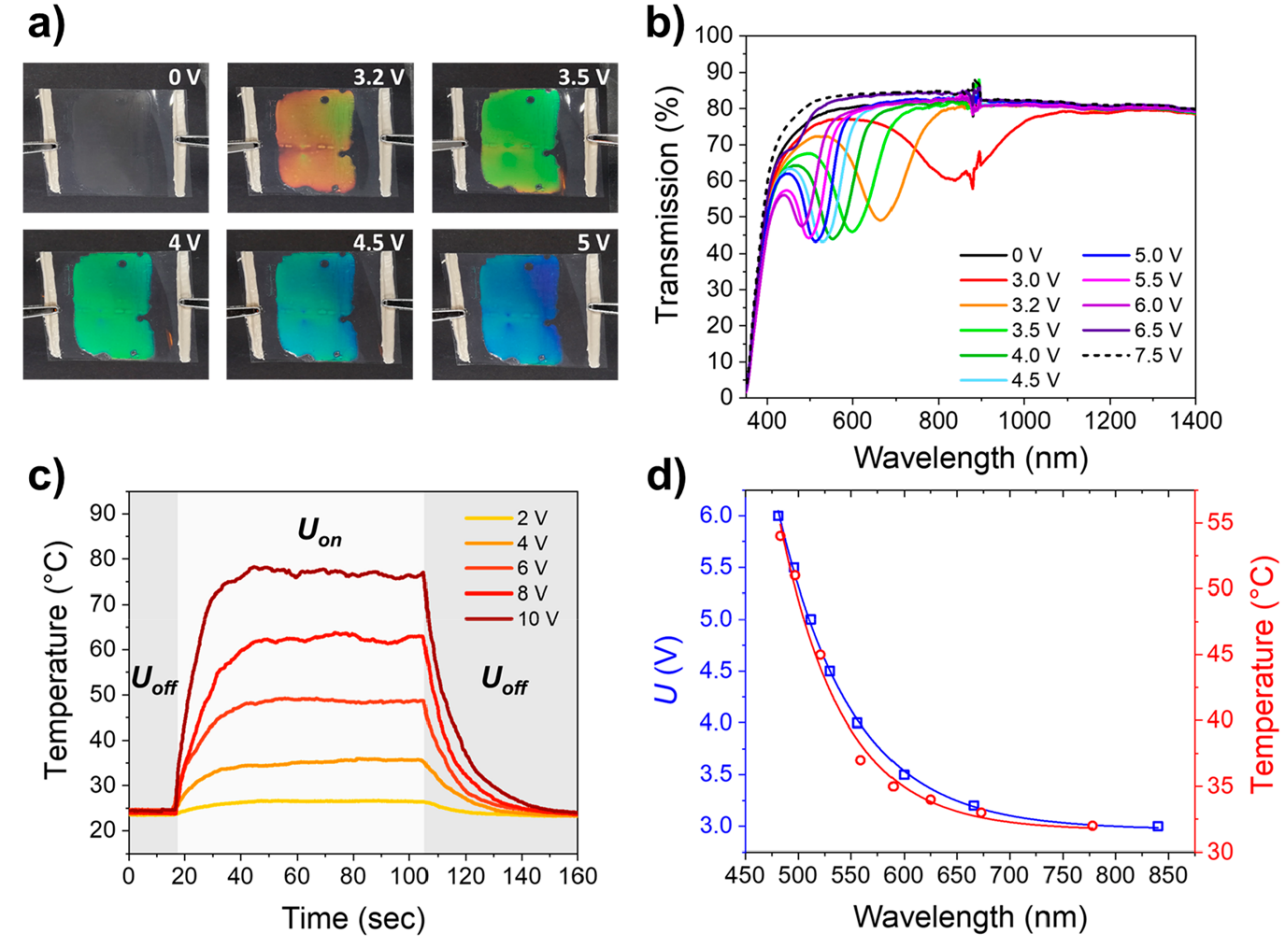
(a) Photographs of the structural colored foil exhibiting electrothermal color tuning with increasing applied potential. (b) Transmission spectra of the structural colored foil, showing the electrothermally induced reflection band shift upon modulating the electrical input. (c) Time-dependent temperature profiles of the photonic coating for different electrical potentials. Joule heating was induced when applying an electrical input (*U*_on_) at *t* = 15 s until it was turned off (*U*_off_) at *t* = 105 s. (d) Comparison of the reflection band shift when varying the electrical potential (blue) or when heating the sample with a hot plate (red).

Electrothermal color tuning of the structural colored foils was investigated by varying the applied potential (Figure 4). A structural color change was noticed over the entire surface area of the coating, implying homogeneous heat transfer from the integrated transparent heater to the photonic coating (Figure 4a). A reflection band shift over the entire visible region was attained at a low potential (*U* = 3–7.5 V), demonstrating energy-efficient color tuning (Figure 4b). The high optical transparency of the foil was confirmed by checking the readability of a black and white sign located at a large distance from the viewer’s eye (Figure S8). By modulating the electrical potential, the reflective color could be tuned. At *U* < 3 V, no reflection band was observed, suggesting mesogenic orientation corresponding to the smectic phase. Increasing the applied potential yielded reflection band shifting analogous to the temperature-induced reflection band shift shown in Figure 3c. Small modifications of the electrical input caused significant color changes, and the reflective color could be adjusted from red to blue by controlling the electrothermal stimulus.

The generated temperature increase was monitored over time to characterize the electrothermal responsiveness of the structural colored foil (Figure 4c). It was observed that the measured *T*_ss_ of the photonic coating was higher than the temperature reported for the AgNW/PET heater at the same applied potential. Most likely, the photonic coating covers the transparent heater, thereby reducing heat losses to the environment. This enhances the induced temperature rise for the photonic coating but slows the kinetics of the electrothermal response compared to the pristine AgNW/PET heater.^55^ The difference in *T*_ss_ between the photonic coating and the uncoated AgNW/PET heater can directly be observed from the recorded infrared images during Joule heating (Figure S9), confirming the difference in heat loss to the environment. No significant deviation of *T*_ss_ was observed when the electrothermal stimulus remained active, proving stable Joule heating performance over time. When analyzing the reflection band shifts under influence of thermal or electrical input, no discrepancy was observed, verifying that the electric current running through the conductive substrate simply generates heat (Figure 4d).

**Figure 5:**
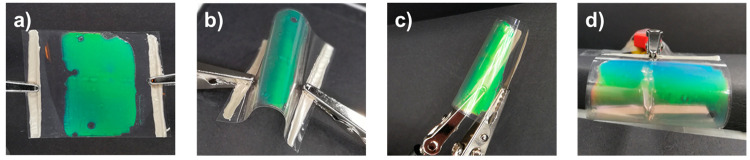
Demonstration of the flexibility of the structural colored foil (*U* = 4 V) while reversibly deforming the system from (a) an unbent to (b) a bent state to (c) deforming the foil until a small bending radius was reached. (d) Front view of the bent electrothermally driven structural colored foil (*U* = 3.5 V) revealing the angular-dependent reflective color.

Lastly, the flexibility and stability of the structural colored foil were investigated when bending the sample multiple times under a continuously applied electrical potential. The reflective color was preserved upon bending and unbending, demonstrating that deforming the foil has no influence on the electrochromic color tuning (Figure 5a–c). The optical response remained constant under influence of cyclic bending stresses thus the integrated film heater and the photonic coating were not affected when being deformed (Figure S10). When looking at the photonic coating from a smaller viewing angle, the reflective color is shifted to lower wavelengths, corresponding to the angular dependency of the CLC layer present in the photonic layer (Figure 5d), confirming the preserved planar CLC alignment upon bending.^56^ By retaining the high transparency and electrothermal color tuning upon bending, this foil has shown features not yet reported for color-tunable electrochromic systems, highlighting its potential for a wide range of photonic applications.

## Conclusion

In this work, we have demonstrated a highly flexible, transparent, structural colored foil that displayed electrochromic color tuning with high optical quality, created with easy processable methods. The photonic foil was colorless at room temperature, but a reflective color appeared autonomously via ambient temperature changes or on-demand when applying an electrical potential. An electrothermal response was induced by the integrated transparent AgNW/PET heater, which could be easily fabricated as a large-area conductive foil by gravure printing. The flexible film heater could induce a rapid temperature increase depending on the applied electrical potential. To establish a temperature-induced photonic response, a structural colored foil was formed out of a CLC ink via PIPS. The photonic coating, consisting of a hard polymerized topcoat protecting a thin thermosensitive CLC layer underneath, could shift its reflection band over the entire visible region at elevated temperatures. The reflective color could be autonomously or manually adjusted by changes in the surrounding temperature or electrical potential, respectively. The optical and electrothermal performances of the developed foil were preserved upon deformation, illustrating its high flexibility and stability. The whole procedure to obtain the structural colored foils can be optimized to a continuous roll-to-roll process, favoring large-area fabrication. Thus, these foils can be incorporated into a wide range of stimuli-responsive photonic devices such as sensors, wearables, and anticounterfeit labels but also could be integrated into large-area applications such as displays and smart windows and be directly installed on top of existing curved surfaces and windows, illustrating its potential for implementation in the automotive industry and building integration.

## Experimental Section

### Synthesis of (*E*)-4,4′-Di(6-methacryloyloxyhexyloxy)-α-methylstilbene (**2**)

A solution of 2.2 mL of methacryloyl chloride (22 mmol) in 20 mL of dichloromethane was added dropwise to a stirred mixture of 4.2 g of (*E*)-4,4′-di(6-hydroxyhexyloxy)-alpha-methylstilbene (**2b**) (10 mmol) (see Figure S11) and 3.0 mL of triethylamine (22 mmol) in 20 mL of dichloromethane at room temperature. After stirring for 16 h, 40 mL of dichloromethane was added, and the solution was extracted subsequently with 50 mL of an aqueous 1 N hydrochloric acid solution and 50 mL of brine. The dichloromethane layer was dried over magnesium sulfate, passed through a 0.5 cm thick silica layer, and evaporated. Finally, 3.2 g of the product (57% yield) was obtained as a white powder with mp = 56 °C after crystallization from ethanol and drying over silica in a vacuum desiccator. ^1^H NMR (400 MHz, δ in ppm, *J* in Hz): 7.44 (d, *J* = 8.8, 2H), 7.27 (d, *J* = 8.8, 2H), 6.89 (d, *J* = 8.8, 2H), 6.88 (d, *J* = 8.8, 2H), 6.71 (s, 1H), 6.10 (s, 2H), 5.48 (p, *J* = 1.6, 2H), 4.16 (t, *J* = 6.6, 4H), 3.98 (t, *J* = 6.4, 4H), 2.24 (d, *J* = 1.5 Hz, 3H), 1.95 (m, *J* = 1.5, 6H), 1.74 (p, *J* = 6.8, 4H), 1.65 (p, *J* = 6.9 Hz, 4H) and 1.50–1.33 (m, 8H). ^13^C NMR (101 MHz, δ in ppm, *: CH or CH_3_, ^#^: CH_2_): 167.69, 158.41, 157.65, 136.66, 135.40, 131.20, 130.42*, 127.06*, 125.91, 125.37^#^, 114.37*, 114.30*, 67.98^#^, 67.93^#^, 64.81^#^, 29.34^#^, 28.72^#^, 25.97^#^, 25.93^#^, 18.49*, and 17.58*. ESI (LC-MS): [M + H]^+^ calculated for C_35_H_47_O_6_^+^: 563.34. Found: 563.33. UV–vis: λ_max_ (dichloromethane) = 296 nm, ε = 28.1 × 10^6^ L mol^–1^ cm^–1^.

### Chemicals

Isobornyl methacrylate (**1**), liquid crystalline mixture MLC-2138, and chiral dopant S811 (**4**) were purchased from Merck and used without any further purification. Irgacure 651 (**3**) was purchased from Ciba Specialty Chemicals Inc.

### Fabrication of the Transparent AgNW/PET Heaters

AgNW/PET substrates were prepared via gravure printing by using an IGT F1 Printability Tester. A silver nanowire (nanowire length and diameter around 30 μm and 30 nm, respectively) containing ink (TranDuctive N15, Genesink) was used for printing, resulting in a transparent film upon drying. Biaxially oriented transparent PET having a thickness of 100 μm (Melinex 506, DuPont Teijin Films) was used as the substrate. The samples were prepared in a ‘gravure printing with pre-inking’ mode while using a printing speed of 0.5 m/s, an anilox force of 250 N, 50% anilox speed, and three pre-ink revolutions for the anilox before the ink was transferred to the PET substrate. After printing, the samples were cured for 5 min at 90 °C in an oven to evaporate the solvent and establish film formation. The described procedure could be repeated multiple times with the same PET foil to lower the sheet resistance of the conductive substrate. The printed AgNW/PET foils were cut into smaller pieces (4 × 4.5 cm^2^),of which the sheet resistance was measured afterward via a four-point probe measurement (T2001A3, Ossila). The reported sheet resistance of the developed heaters stemmed from the average value and corresponding variance that were deducted from at least 10 measured sheet resistance values.

### Temperature-Responsive Photonic Coating

First, 44.5 wt % monoacrylate (**1**), 5 wt % dimethacrylate (**2**), 0.5 wt % photoinitiator (**3**), 15 wt % chiral dopant (**4**), and 35 wt % MLC-2138 were added together and stirred at 40 °C until a homogeneous mixture was obtained. The mixture (80 μL) was bar coated on top of a gravure printed AgNW/PET substrate (4 × 4.5 cm^2^) at room temperature. A wire-wound rod, having an 80 μm gap height, was pushed over the substrate to spread the mixture over the substrate area. Photopolymerization was performed by using an EXFO Omnicure S2000 Mercury Lamp. The coatings were cured at 54 °C in a nitrogen environment at 1.5 mW/cm^2^ for 20 min. Afterward, postcuring was carried out at 20 mW/cm^2^ for 5 min.

### Analysis of the Electrothermal Response

The electrothermal response of the developed foils (4 × 4.5 cm^2^) was generated by applying an electrical potential. Before inducing the electrothermal response, a conductive epoxy glue (Chemtronics, CW2400) was spread out over the width of the films on both sides of the heater and cured at 70 °C for 30 min to establish proper electrical contact with the crocodile clips, connected to a direct current (DC) power supply (Keithley 2400 SourceMeter). The induced electrothermal response was studied when modifying the electrical potential. The time–temperature profiles and infrared images were recorded by a high-speed thermal camera (Gobi, Xenics), which tracks the film’s surface temperature over time.

### Characterization of the Structural Colored Foils

Transmission spectra were performed at room temperature and measured by a Shimadzu UV-3102 PC UV/vis/NIR spectrophotometer equipped with an MPC-3100 sample compartment. A Linkam TMS93/LMP93 temperature control stage was installed in the spectrophotometer to analyze the thermal response of the photonic foil, while a DC power supply (Keithley 2400 SourceMeter) was used to study the electrothermal behavior. When changing the temperature or electrical potential between measurements, a waiting time of 2 min was respected before recording the transmission spectra to establish steady-state conditions. The noise observed around 860 nm in all the measurements was due to a detector change. The transmission spectra were recorded when using air as a baseline.

Analysis of the coating morphology was carried out by preparing cross sections of the photonic system (coating and substrate) by cryo-microtoming (Leica EM UC7). All morphology measurements were recorded at room temperature. Optical microscopy (Keyence VHX-5000) images were recorded in bright-field mode. Atomic force microscopy (Bruker Dimension FastScan) images were obtained in tapping mode with a scan rate of 1–4 Hz. Raman spectroscopy was performed by a Bruker SENTERRA dispersive Raman microscope. A confocal Raman analysis was executed by measuring a confocal line scan, starting from the coating surface to the substrate, using a 532 nm laser, 100× objective, and 25 scans per step. The confocal step size was 5 μm and the measurement resolution was ∼20 μm.

## Supporting Information Available

Electrothermal heating of the gravure printed AgNW/PET substrate, cyclic bending test of the AgNW/PET substrate, AgNW density on top of the gravure printed AgNW/PET foils, phase behavior of the CLC mixture, smectic-cholesteric phase transition and corresponding color change of the photonic coating, temperature-induced reflection band shift of the photonic coating on top a pristine PET substrate, electrothermal color tuning of the structural colored foil showing high optical quality, infrared image of the structural colored foil demonstrating the Joule heating performance, electrochromic response of the structural colored foil upon cyclic deformation, synthesis and characterization of (*E*)-4,4′-di(6-methacryloyloxyhexyloxy)-α-methylstilbene (**2**) (PDF)

## Abbreviations

AgNWs: silver nanowires
CLC: cholesteric liquid crystal
PIPS: photoinduced phase separation
PET: polyethylene terephthalate
*U*: electrical potential
*R*_s_: sheet resistance
*T*_ss_: steady-state temperature
AFM: atomic force microscopy
