# Supplementary material for: Ink-Deposited Transparent Electrochromic Structural Colored Foils
Source: ACS Appl Mater Interfaces. 2022 Aug 19;14(34):39375–83. doi: 10.1021/acsami.2c11106 (PMC9437895; doi:10.1021/acsami.2c11106)
Supplement: Supplementary file 1 — am2c11106_si_001.pdf [file am2c11106_si_001.pdf]

SUPPORTING INFORMATION

Ink-deposited transparent electrochromic structural colored foils

Arne A. F. Froyen[1,3], Nadia Grossiord[1,2], Jos de Heer[2], Toob Meerman[2], Lanti Yang[2], Johan Lub[1], Albert P. H. J. Schenning[1,3,4,]*

[1] Stimuli-responsive Functional Materials and Devices, Department of Chemical Engineering and Chemistry, Eindhoven University of Technology, P.O. Box 513, 5600 MB, Eindhoven, The Netherlands

[2] SABIC, Plasticslaan 1, 4612 PX, Bergen op Zoom, The Netherlands

[3] Institute for Complex Molecular Systems, Eindhoven University of Technology, Den Dolech 2, 5600 MB, Eindhoven, The Netherlands

[4] SCNU-TUE Joint Laboratory of Device Integrated Responsive Materials (DIRM), South China Normal University, Guangzhou Higher Education Mega Center, 510006, Guangzhou, China

[*]Corresponding author, email: A.P.H.J.Schenning@tue.nl

Electrothermal heating of the gravure printed AgNW/PET substrate

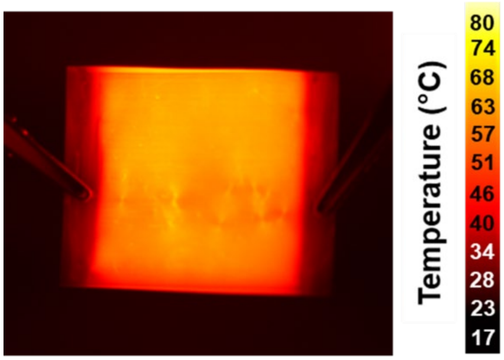

Figure S1. Recorded infrared image of the AgNW/PET heater at T_{ss} for U = 10 V with corresponding temperature scale, showing homogeneous heating over the entire substrate area.

Cyclic bending test of the AgNW/PET substrate

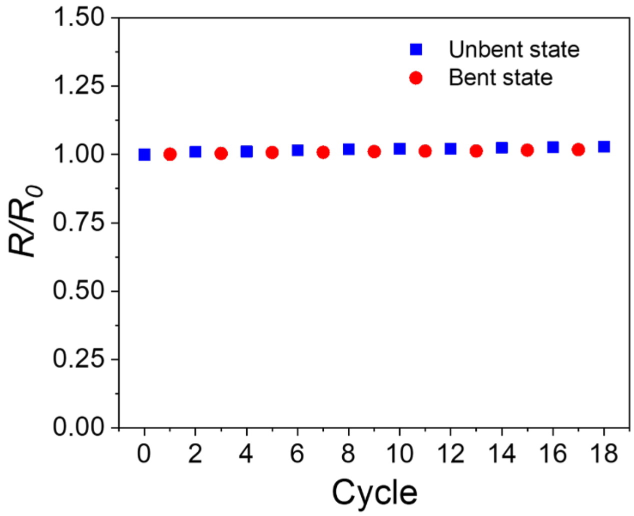

Figure S2. Cyclic bending test of the printed AgNW/PET heater, showing a stable performance under repetitive deformation. The ratio of the measured resistance to the initial resistance (R/R_0) was reported upon bending (bending radius = 3 mm) and unbending the sample.

AgNW density on top of the gravure printed AgNW/PET foils

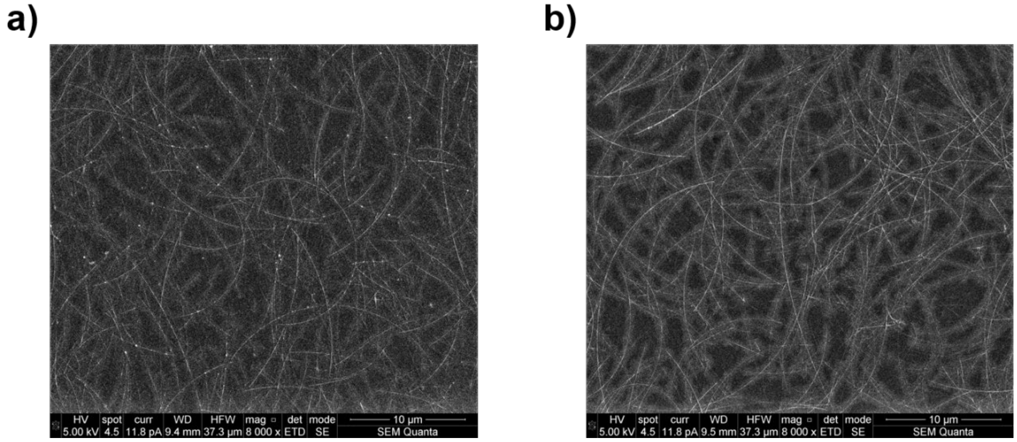

Figure S3. Scanning electron microscopy images of AgNW/PET foils after (a) a single printing step, (b) three subsequent printing steps.

Phase behavior of the CLC mixture

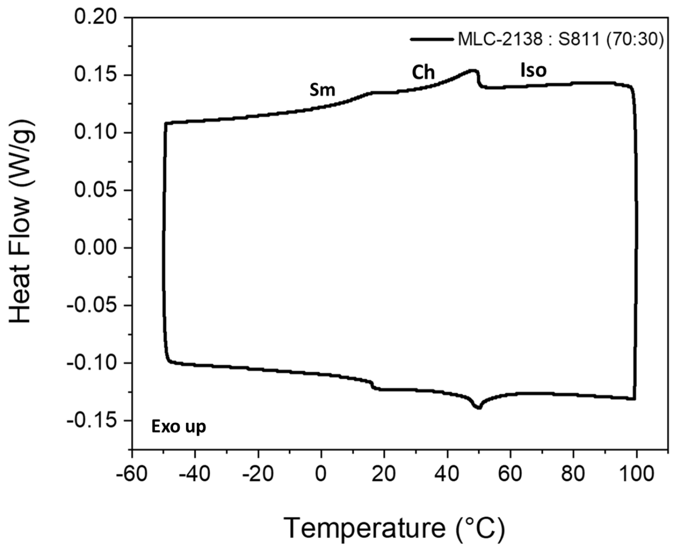

Figure S4. DSC measurement of the pure CLC mixture (70 wt % MLC-2138, 30 wt % S811), showing the smectic-cholesteric and cholesteric-isotropic phase transitions. The measurement was performed at a heating/cooling rate of 3 °C/min.

Smectic-cholesteric phase transition and corresponding color change of the photonic coating

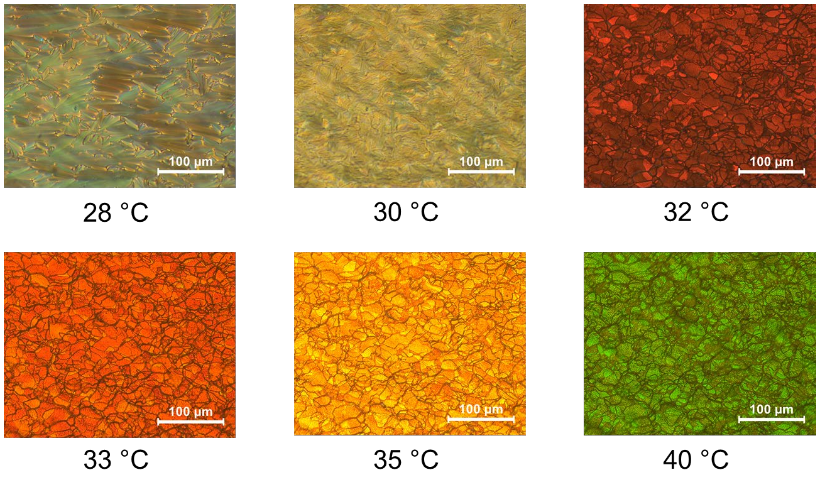

Figure S5. Polarized optical microscopy images (transmission mode with crossed polarizers) of the structural colored coating, featuring the temperature-responsive color change that is caused by the smectic-cholesteric phase transition.

Temperature-induced reflection band shift of the photonic coating on top a pristine PET substrate

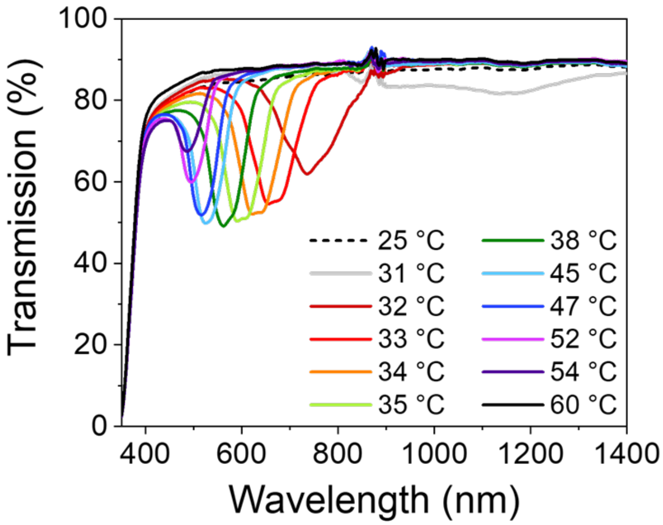

Figure S6. Transmission spectra showing the temperature-responsive reflection band shift of the phase-separated photonic coating on top of a pristine transparent PET substrate when being heated by a hot plate. Air was used as a baseline.

Confocal Raman spectra of pure monomer and CLC mixtures

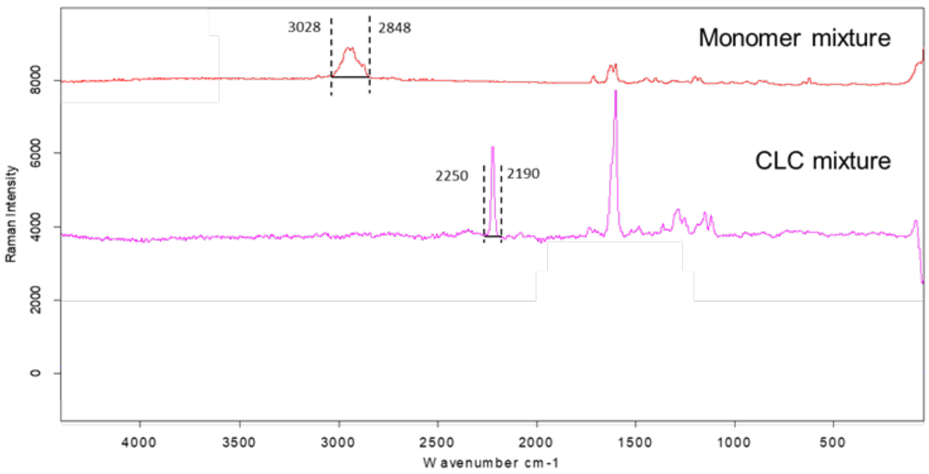

Figure S7. a) Confocal Raman spectra of the pure monomer and CLC mixture. The ratio between two distinct peak areas (2190-2250 cm^{-1} and 2848-3028 cm^{-1}) was used for the composition analysis.

Electrothermal color tuning of the structural colored foil showing high optical quality

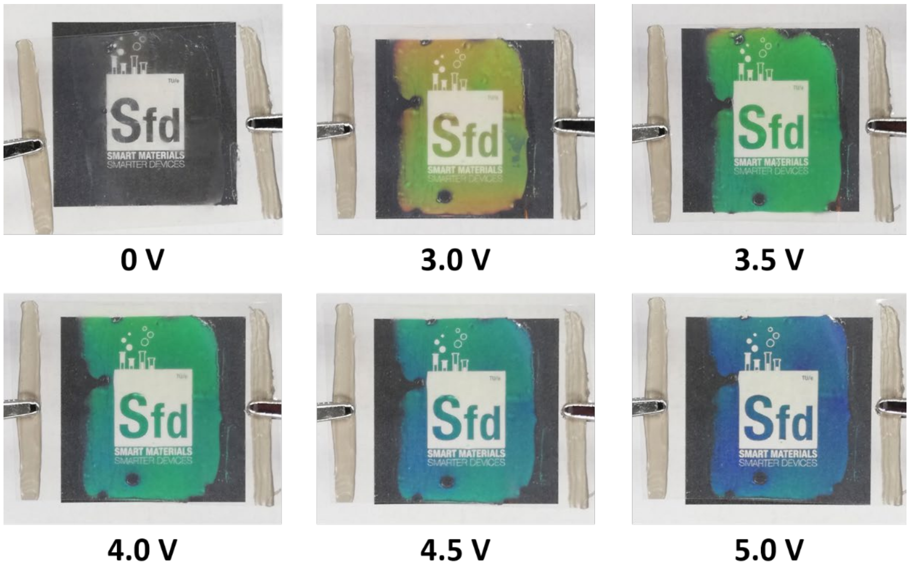

Figure S8. Electrothermal color tuning of the structural colored foil when enhancing the voltage amplitude. A black and white background was used, which could be read from a larger distance, highlighting that high optical quality was achieved. The sample-background distance was 5 cm, and the camera-sample distance was 15 cm.

Infrared image of the structural colored foil demonstrating the Joule heating performance

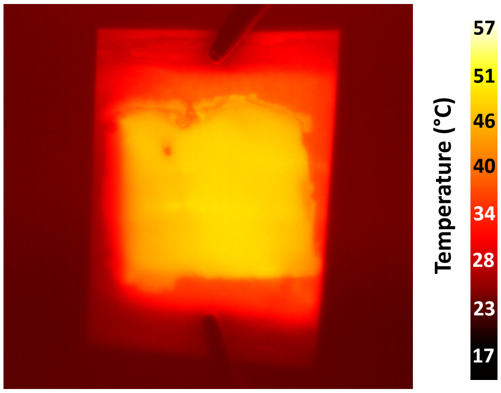

Figure S9. Recorded infrared image of the photonic coating on top of the AgNW/PET heater upon applying a DC voltage ($U = 6$ V). A waiting time of 120 sec was respected to measure under steady-state conditions.

Electrochromic response of the structural colored foil upon cyclic deformation

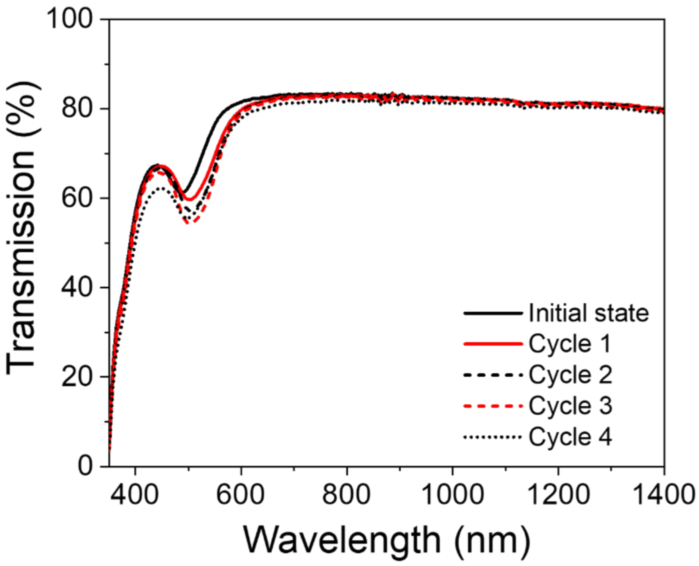

Figure S10. Transmission spectra of an unbent photonic foil driven by an electrothermal stimulus ($U = 5$ V). After each transmission measurement/cycle, the sample was bent to a bending radius of 4 mm and unbent again to a flat state while a DC voltage was applied.

Synthesis and characterization of (*E*)-4,4'-di-(6-methacryloyloxyhexyloxy)-α-methylstilbene (2)

(*E*)-4,4'-di-(6-methacryloyloxyhexyloxy)-α-methylstilbene **2 (**see scheme 1) was synthesized by methacrylation of diol **2b** which in turn was made by condensation of phenyl ether derivative **2a** with chloroacetone followed by a [1.3] carbon shift of the intermediate chlorobisphenol-A derivative according to a procedure described for a similar reaction with anisole.[1,2]

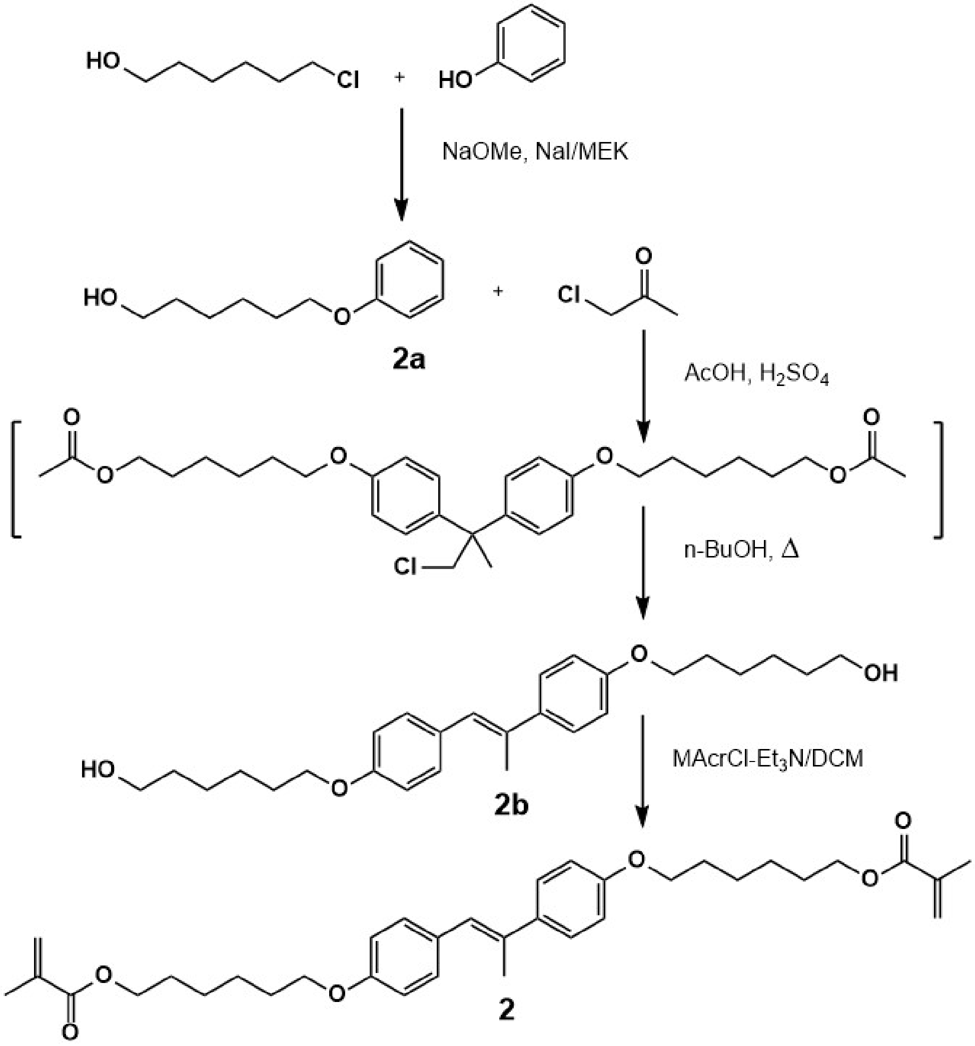

Figure S11. Synthesis of (*E*)-4,4'-di-(6-methacryloyloxyhexyloxy)-α-methylstilbene (**2**).

Synthesis of (6-hydroxyhexyloxy)benzene (**2a**).

27 g of powdered sodium methanolate (0.5 mol) was added in portions to a vigorously stirred solution of 47 g of phenol (0.5 mol) in 100 ml of butanone under nitrogen. After complete dissolution, 15 g of sodium iodide (0.1 mol) and 68 ml of 6-chlorohexanol (0.5 mol) were added and the solution was refluxed for 16 hours. After cooling, filtration, and evaporation, 150 ml of diethyl ether and 100 ml of a 1.2 N aqueous sodium hydroxide solution were added. After separation, the diethyl ether layer was extracted with 100 ml of brine, dried over magnesium sulfate and evaporated. 73 g of the product (75% yield) was obtained as a slowly crystallizing oil after fractionation (bp = 106°C at 0.13 mb). ^{1}H-NMR (400 MHz, δ in ppm, J in Hz): 7.29 (m, J_1 = 7.4, J_2 = 8.8, 2H), 6.92 (t, J = 7.4, 1H), 6.89 (d, J = 8.6, 2H), 3.94 (t, 2H, J = 6.4), 3.61 (t, 2H, J = 6.8), 2.5-3.0 (br, 1H), 1.85 (p, 2H, J = 6.6) and 1.38 – 1.75 (m, 6H). ^{13}C-NMR (101 MHz, δ in ppm, *: CH or CH_3, $^\#$: CH_2): 159.0, 129.4*, 120.6*, 114.5*, 67.9$^\#$, 62.4$^\#$, 33.9$^\#$, 32.7$^\#$, 29.3$^\#$ and 25.6$^\#$.

Synthesis of (*E*)-4,4'-di-(6-hydroxyhexyloxy)-α-methylstilbene (**2b**).

A mixture of 14.8 ml of sulfuric acid (0.27 mol) and 8 ml of acetic acid (0.14 mol) was added dropwise to a mechanically stirred mixture of 39 g of (6-hydroxyhexyloxy) benzene **2a** (0.2 mol) and 8.0 ml of chloroacetone (0.1 mol) cooled in an ice bath, at such a rate that the temperature didn't exceed 15°C. After stirring for another 16 hours at room temperature 200 ml of ethyl acetate was added followed by 300 ml of water. After separation, the organic layer was extracted subsequently with 200 ml of a 10% aqueous sodium bicarbonate solution and 200 ml of brine. The crude acetylated bisphenol intermediate, obtained after drying over magnesium sulfate and evaporation, was mixed with 150 ml of n-butanol, and refluxed for 16 hours. Upon cooling, the product precipitates. 12.4 g of the product (30% yield) was obtained after crystallization from

methanol and drying over silica in a vacuum desiccator. ^{1}H-NMR (400 MHz, δ in ppm, J in Hz): 7.43 (d, J = 8.8, 2H), 7.28 (d, J = 8.8, 2H), 6.89 (d, J = 8.8, 2H), 6.88 (d, J = 8.8, 2H), 6.72 (s, 1H), 4.00 (t, J = 6.4, 4H), 3.65 (t, J = 6.6, 4H), 3.0-4.0 (br, 2H) 2.25 (d, J = 1.3 Hz, 3H), 1.75 (p, J = 6.8, 4H) and 1.55 – 1.33 (m, 12H). ^{13}C-NMR (101 MHz, δ in ppm, *: CH or CH_3, $^{\#}$: CH_2): 158.41, 157.65, 135.40, 131.20, 130.42*, 127.06*, 125.91, 114.37*, 114.30*, 67.98$^{\#}$, 67.93$^{\#}$, 63.32$^{\#}$, 33.11$^{\#}$, 29.71$^{\#}$, 26.39$^{\#}$, 25.94$^{\#}$ and 17.58*.

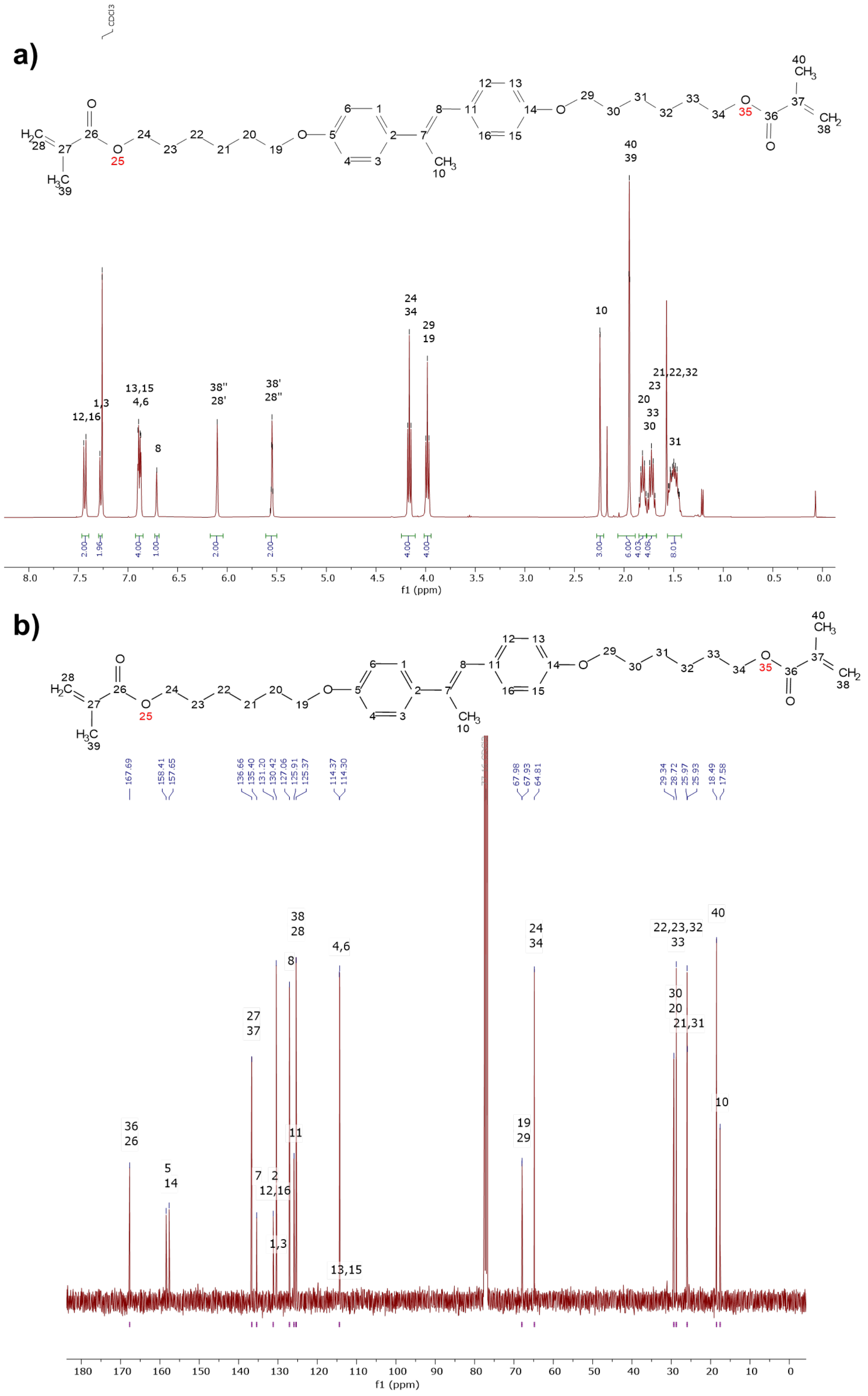

Figure S12. NMR spectra of **2**. a) ^{1}H NMR spectra. b) ^{13}C NMR spectra.

REFERENCES

(1) Zaheer S. H.; Singh, B.; Bhargava P. M.; Kacker I. K.; Ramachandran K.; Sastri V. D. N.; Rao N. S. Reactions of α-Halogeno-ketones with Aromatic Compounds. Part I. Reactions of Chloroacetone and 3-Chlorobutanone with Phenol and its Ethers *J. Chem. Soc.* **1954**, 3360–3362. https://doi.org/10.1039/JR9540003360

(2) Scholz, M.; Ulbrich, H. K.; Soehnlein, O.; Lindbom, L.; Mattern, A.; Dannhardt, G. Diaryl-Dithiolanes and -Isothiazoles: COX-1/COX-2 and 5-LOX-Inhibitory, •OH Scavenging and Anti-Adhesive Activities. *Bioorganic Med. Chem.* **2009**, *17* (2), 558–568. https://doi.org/10.1016/j.bmc.2008.11.074.
